# Retaining red bell pepper quality by perforated compostable packaging

**DOI:** 10.1002/fsn3.2329

**Published:** 2021-05-25

**Authors:** Abiola Owoyemi, Victor Rodov, Ron Porat

**Affiliations:** ^1^ Department of Postharvest Science ARO The Volcani Center Rishon LeZion Israel; ^2^ The Robert H Smith Faculty of Agriculture, Food and Environment The Hebrew University of Jerusalem Rehovot Israel

**Keywords:** compostable, packaging, pepper, plastic, postharvest

## Abstract

Retail packages are widely used to preserve pepper fruit quality. However, due to the negative impact of conventional plastics on the environment there is an urgent need to replace these packaging materials with recyclable or compostable alternatives. Hereby, we evaluated the effects of compostable modified atmosphere packages with different perforation rates on keeping the quality of red bell pepper fruit during extended shelf life and simulated supply chain conditions. The results indicated that micro‐perforated (µP) compostable packages creating an atmosphere of 15%–18% O_2_ and 2%–5% CO_2_, as well as macro‐perforated (MP) compostable packages creating an atmosphere of 20%–21% O_2_ and 0.1%–0.5% CO_2_, effectively retained red bell pepper quality by reducing fruit weight loss, shriveling and softening, and by retaining flavor acceptance and visual appearance. On the other hand, nonperforated compostable packages resulted in the creation of anaerobic conditions (O_2_ < 1% and CO_2_ > 9%), and harmed produce quality as manifested by enhanced softening, decay, peel damage, and off‐flavors. Overall, µP and MP compostable packages extended pepper fruit shelf life from 1 week to 3 weeks under continuous shelf life conditions, and from 2 weeks to 4 weeks under simulated supply chain and refrigerated storage conditions.

## Graphical abstract

The findings observed in this study indicate that perforated compostable packages were very effective in retaining red bell pepper fruit quality after harvest, and therefore may provide a recommended environmentally friendly alternative to replace the common usage of conventional retail plastic packages.

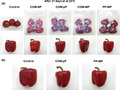

## 1 INTRODUCTION

Fruit and vegetables are perishable produce (Kader, 2002). The main factors limiting the postharvest storage capability of bell pepper fruit are water loss, shriveling, softening, and development of decay and chilling injuries (Cantwell, 2002).

A key technology to preserve fruit and vegetables freshness after harvest is the use of packaging that creates beneficial modified atmosphere (MA) and modified humidity (MH) environments that retains freshness, inhibit ripening and senescence, and reduces decay development and appearance of physiological disorders (Kader et al., 1989; Rai et al., 2002). The optimal MA and MH conditions within modified atmosphere packages vary depending on produce types, their physiological characteristics, respiration rates and tolerances to low O_2_ and high CO_2_ gas atmospheres (Kader, 2002; Saltveit, 2003). In this respect, it was reported that bell pepper fruit could not tolerate O_2_ levels below 2%, which enhance ethanol fermentation, and CO_2_ levels above 5%, which enhance pitting, discoloration and softening (Cantwell, 2002; Saltveit, 2003).

Several studies previously indicated that modified atmosphere packaging retains pepper fruit quality and extends its postharvest storage life mainly by alleviating water stress as well as by reducing the development of decay and chilling damage (Akbudak, 2008; Ben‐Yehoshua et al., 1983; Chitravathi et al., 2015; Meir et al., 1995; Wall & Berghage, 1996). For example, Singh et al. (2014) reported that MA packaging extended the shelf life of bell peppers under continuous storage at 8°C from 21 to 42 days, and Chitravathi et al. (2015) reported that different modified atmosphere packages extended the shelf life of chili peppers under continuous storage at 8°C from 15 up to 28 days. Singh et al. (2014) and Devgan et al. (2019) reported that further extension of the storage life of bell peppers could be reached by using active modified atmosphere packages with the addition of moisture and O_2_ absorbers.

Despite the great advantages of MA packages in preserving the freshness and quality of fruit and vegetables, single‐use plastics are responsible for massive environmental pollution, especially of rivers and oceans (Xanthos & Walker, 2017). Therefore, the New Plastics Economy Global Commitment, already signed by more than 850 international companies, have set a goal that by 2025, all plastic packaging will be either reusable, recyclable or compostable (Ellen MacArthur & UNEP, 2019). In a previous study, Ornelas‐Paz et al. (2012) has examined the efficacy of using recycled low‐density polyethylene packages in order to retain the postharvest quality of Jalapeno peppers. The aim of the current study was to examine the effects of compostable packages with different perforation rates on retaining postharvest quality of red bell peppers during continuous shelf life and simulated marketing conditions. The use of compostable materials may provide an environmentally friendly alternative that may replace the current use of petroleum‐based plastic packages, depending that the compostable packages will indeed be as effective as conventional plastic packages in retaining produce freshness and quality (Khalil et al., 2018; Owoyemi et al., 2021; Wróblewska‐Krepsztul et al., 2018). Furthermore, unlike previous studies that mainly evaluated the effects of modified atmosphere packages under optimal storage temperatures of 7–8°C, we hereby examined their effects under simulated real‐life conditions including extended shelf life, and simulated supply chain conditions.

## 2 MATERIAL AND METHODS

### 2.1 Plant material

Red bell peppers (*Capsicum* *annuum*) were harvested on 12 February 2020 from a commercial greenhouse in Moshav Coach, Israel. The fruit were transferred within 1 hr to the ARO, the Volcani Center, where they were sorted for a uniform red color and clarify of defects.

### 2.2 Packaging treatments

Part of fruit remained unpacked as a control, while the other fruit were packed in nonperforated (NP), micro‐perforated (µP), and macro‐perforated (MP) compostable packages or in MP polypropylene (PP) packages. The compostable packages were purchased from TIPA^®^ Corp. (Hod HaSharon, Israel), and the PP packages were purchased from R.O.P. Ltd. (Hahotrim, Israel). All packages were 35 µm thick and in the size of 30 × 40 cm. The oxygen transmission rates (OTR) of the compostable and PP films were 875 and 2,019 cc/m^2^/day, respectively, and the water vapor transmission rates (WVTR) were 40.8 and 8.1 gr/m^2^/day, respectively. Micro‐perforations were conducted by making 8 holes with a 0.5‐mm needle (PIC Ago Ipodermico, 25G hypodermic needle, Grandate, Italy), and macro‐perforations were conducted by making 8 holes of 6 mm width using an office paper hole puncher. The packages were sealed using an impulse heat sealer (Swery Electronics Ltd., Petah Tikva, Israel). Each treatment included 24 packages with four fruit in each package, and three packages were inspected at each evaluation point.

### 2.3 Postharvest storage

Half of the fruit were continuously stored at 22°C, in order to simulate extended shelf life conditions, while the other half were stored for 2 days at 15°C + 2 days at 22°C for simulation of retail storage and marketing, and 2, 4, or 6 weeks of refrigerator storage at 4°C for simulation of home storage conditions.

### 2.4 Gas measurements

The O_2_ and CO_2_ concentrations in the packages headspaces were measured using an OxyBABY gas analyzer (WITT Gasetechnik GmbH & Co KG, Witten, Germany).

### 2.5 Quality measurements

Pepper fruit quality was evaluated at the beginning of the experiments and during continuous shelf life and simulated supply chain conditions. Fruit in the continuous shelf life treatment was evaluated and after 1, 2, 3, and 4 weeks at 22°C, while fruit in the simulated supply chain treatment were evaluated after 2 days of storage and distribution at 15°C and 2 days of marketing at 22°C, plus 2, 4, and 6 weeks of home‐refrigerated storage at 4°C.

Fruit weight loss was evaluated by weighing the fruit in each treatment before and after storage. Fruit shriveling, softening, and peel damage indices were provided according to a visual score of between 0 till 3, in which 0 = *none*, 1 = *slight*, 2 = *moderate,* and 3 = *severe*. Decay levels were recorded as the percentage of infected fruit among the total amount of fruit in each package. Flavor scores were provided according to a 9‐point hedonic scale, in which 1 = *very bad* and 9 = *excellent*. At last, an overall acceptance score for each package was provided using a 5‐point scale, in which 1 = *very bad*, 2 = *poor*, 3 = *fair*, 4 = *good,* and 5 = *excellent*; an acceptance score of 2.5 represented the minimum quality acceptability for human consumption. Flavor and acceptance evaluations were provided by 3 panelists.

### 2.6 Statistical analysis

Microsoft Office Excel was used to calculate means and standard errors. Analysis of variance (ANOVA) was conducted by the aid of the JMP statistical software, version 14 (SAS Institute Inc., Cary, NC, USA).

## 3 RESULTS

### 3.1 Visual appearance

At the beginning of the experiment, all fruit were red, firm, and free of blemishes. However, during storage the fruit deteriorated, lost weight, and shriveled.

**FIGURE 1:**
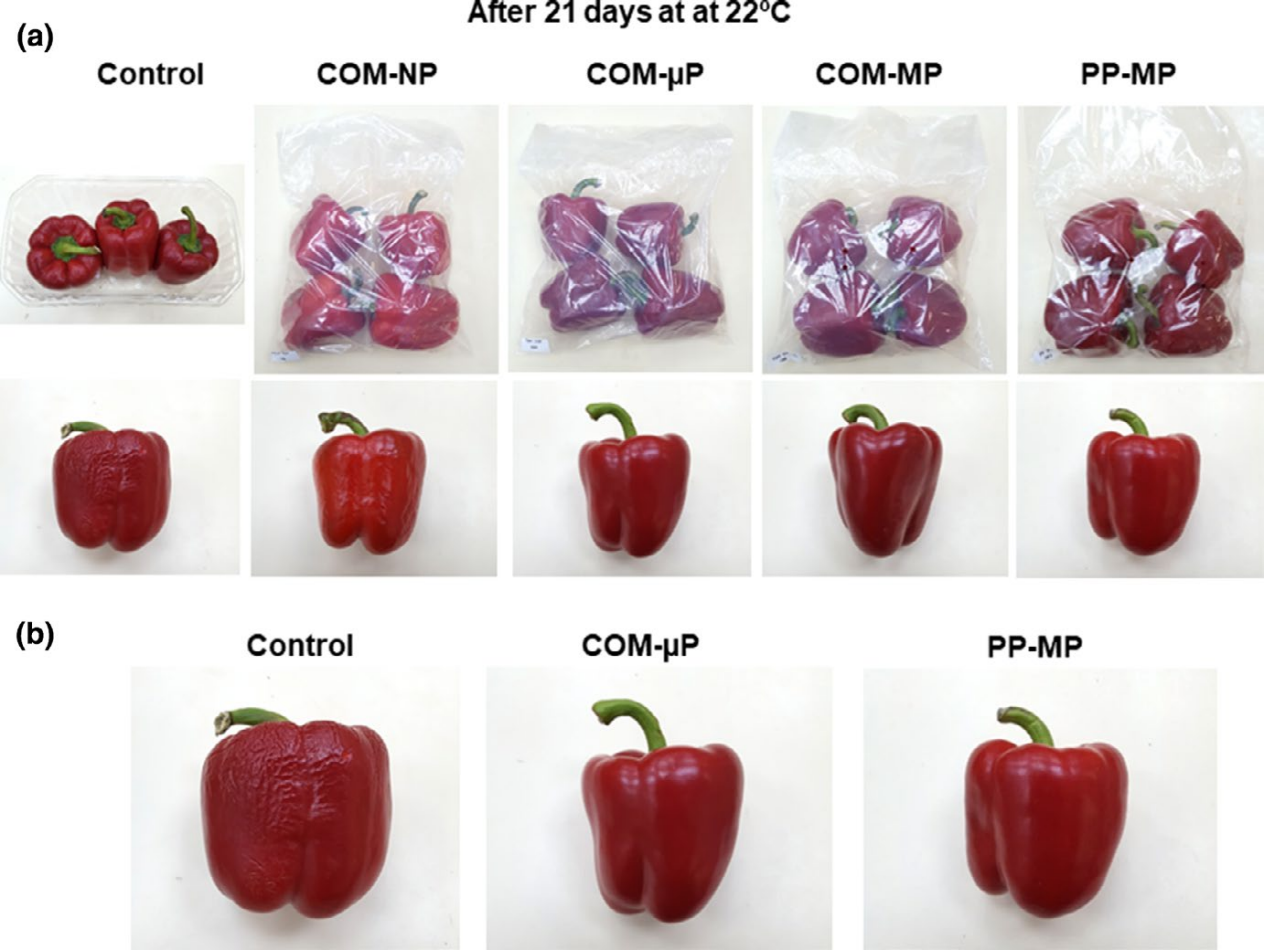
Photographs of red bell peppers stored in different packages for 3 weeks at shelf conditions at 22°C. (a) Photographs of packages and single fruit, and (b) enlarged pictures of fruit from the control, COM‐µP and PP‐MP treatments. µP, micro‐perforated; COM, compostable; MP, macro‐perforated; NP, nonperforated; PP, polypropylene

**FIGURE 2:**
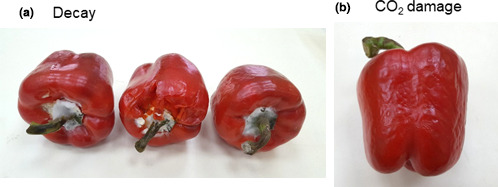
Photographs of red bell peppers stored in NP compostable packages for 4 weeks at shelf conditions at 22°C. The picture at the left shows decay development and the picture on the right shows CO_2_ damage

After 3 weeks of extended shelf life at 22°C, the control untreated peppers lost weight and suffered from shriveling (Figure 1). In contrast, the peppers within the µP and MP compostable and PP packages remained smooth, firm and had a fresh appearance (Figure 1). Peppers stored in the NP compostable packages suffered from decay and peel pitting (Figure 2).

**FIGURE 3:**
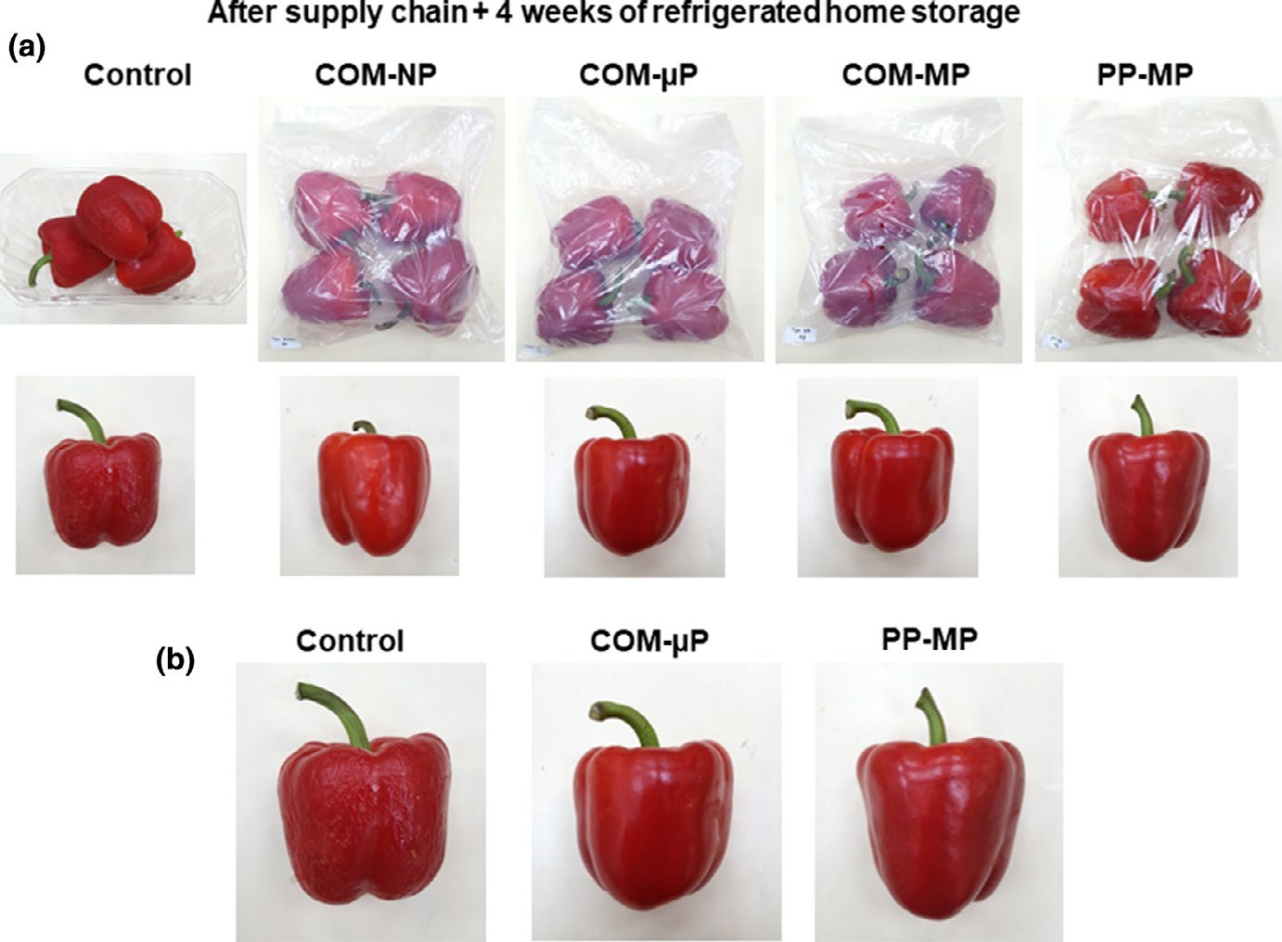
Photographs of red bell peppers stored in different packages under simulated supply chain conditions including 2 days at 15°C and 2 days at 22°C followed by 4 weeks of refrigerated storage at 4°C. (a) Photographs of packages and single fruit, and (b) enlarged pictures of fruit from the control, COM‐µP and PP‐MP treatments. µP, micro‐perforated; COM, compostable; MP, macro‐perforated; NP, nonperforated; PP, polypropylene

After the simulated supply chain and 4 weeks of refrigerated home storage at 4°C, the control fruit lost weight and suffered from severe shriveling (Figure 3). In contrast, the peppers within the µP and MP compostable and PP packages yet remained smooth, firm and had a good and attractive appearance (Figure 3). Peppers stored in the NP compostable packages remained after 4 weeks of refrigerated home storage at a fair quality, but suffered from slight peel damage (Figure 3).

### 3.2 Atmosphere compositions

**FIGURE 4:**
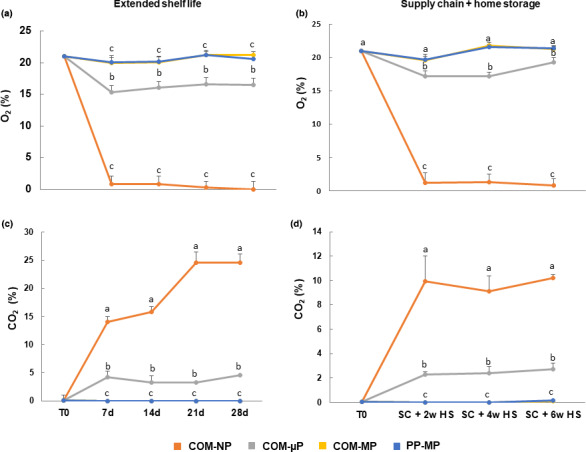
O_2_ and CO_2_ levels in the headspaces of different packages of red bell pepper fruit. Measurements were taken during extended shelf at 22°C, and after a simulated supply chain treatment including 2 days at 15°C and 2 days at 22°C followed by up to 6 weeks of refrigerated storage at 4°C. Data are means ± *SE* of 3 replications. Different letters indicate significant differences at *p* ≤ .05. µP, micro‐perforated; COM, compostable; MP, macro‐perforated; NP, nonperforated; PP, polypropylene

After 1 week under shelf life conditions, and after the simulated supply chain and 2 weeks of refrigerated storage at 4°C, the O_2_ levels in the NP compostable packages were extremely low (<1.0%) and the CO_2_ levels increased to above 10% (Figure 4). Afterwards, the O_2_ levels in the NP compostable packages further decreased to 0% under shelf life conditions and to 0.7% under the refrigerated storage conditions, and the CO_2_ levels increased till 24.6% under shelf life conditions and remained at 9%–10% under the refrigerated home storage conditions (Figure 4). The O_2_ levels in the µP packages moderately decreased to just 15%–16% under shelf life conditions and till 17%–19% under the refrigerated storage conditions, while the CO_2_ levels moderately increased to 3.2%–4.6% under shelf life conditions and till 2.3%–2.7% under the refrigerated storage conditions (Figure 4). The O_2_ and CO_2_ levels in the MP packages were more or less similar to those of regular air (Figure 4).

### 3.3 Water loss, shriveling and softening

**FIGURE 5:**
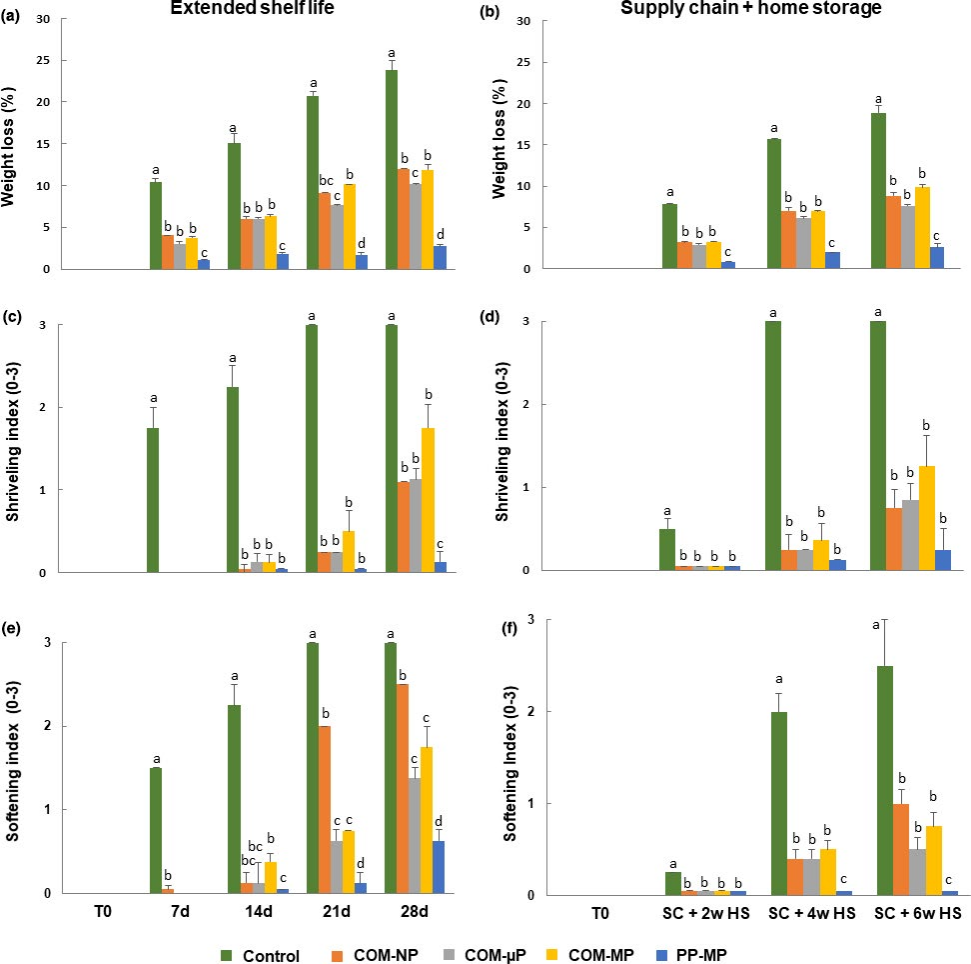
Weight loss levels, and shriveling and softening indices of red bell pepper fruit in different packages. Measurements were taken during extended shelf at 22°C, and after a simulated supply chain treatment including 2 days at 15°C and 2 days at 22°C followed by up to 6 weeks of refrigerated storage at 4°C. Data are means ± *SE* of 3 replications. Different letters indicate significant differences at *p* ≤ .05. µP, micro‐perforated; COM, compostable; MP, macro‐perforated; NP, nonperforated; PP, polypropylene

A main problem in postharvest storage of pepper fruit is excessive water loss. The results indicate that control fruit lost nearly 10%, 15%, 20%, and 24% of their initial weight after 1, 2, 3, and 4 weeks of storage under shelf life conditions, respectively, and nearly 8%, 16% and 18% of their initial weight following the simulated supply chain plus 2, 4, and 6 weeks of refrigerated storage, respectively (Figure 5a,b). In distinction, peppers in the various compostable packages lost significantly less weight (nearly half) regarding the levels observed in control nonpacked fruit. At the end of the 4‐week extended shelf life period, the peppers in the compostable packages lost between 10% and 12% of their initial weight as compared with 24% in the control fruit. At the end of the simulated supply chain and 6 weeks of refrigerated storage, the peppers in the compostable packages lost between 8% and 10% of their initial weight as compared with 19% in the control fruit (Figure 5a,b). The fruit in the PP packages lost significantly less weight. At the end of the 4 weeks extended shelf life period, the peppers in the PP packages lost only 2.75% of their initial weight, and after the simulated supply chain and 6 weeks of refrigerated home storage, they lost just 2.6% of their initial weight (Figure 5a,b).

In accordance with the observed weight loss levels, the control nonpacked fruit suffered from severe shriveling which became significantly evident already after 1 week under extended shelf life at 22°C, and after 2 and especially 4 weeks of the simulated marketing and refrigerated storage (Figure 5c,d). In distinction, the peppers in the various compostable packages began to suffer from meaningful shriveling only after 4 weeks of extended shelf life and 6 weeks of refrigerated storage (Figure 5c,d). The peppers kept in the PP packages barely suffered from shriveling.

Along with the appearance of shriveling symptoms, the control nonpacked fruit also became significantly and meaningfully softer already after 1 week under extended shelf life at 22°C, and after 2 and especially 4 weeks of simulated marketing and refrigerated storage (Figure 5e,f). Interestingly, the fruit in the NP compostable packages became significantly soft after 3 weeks under extended shelf life, while the fruit in µP and MP compostable packages became soft only after 4 weeks (Figure 5e,f). The fruit in PP packages remained significantly firmer.

### 3.4 Decay

**FIGURE 6:**
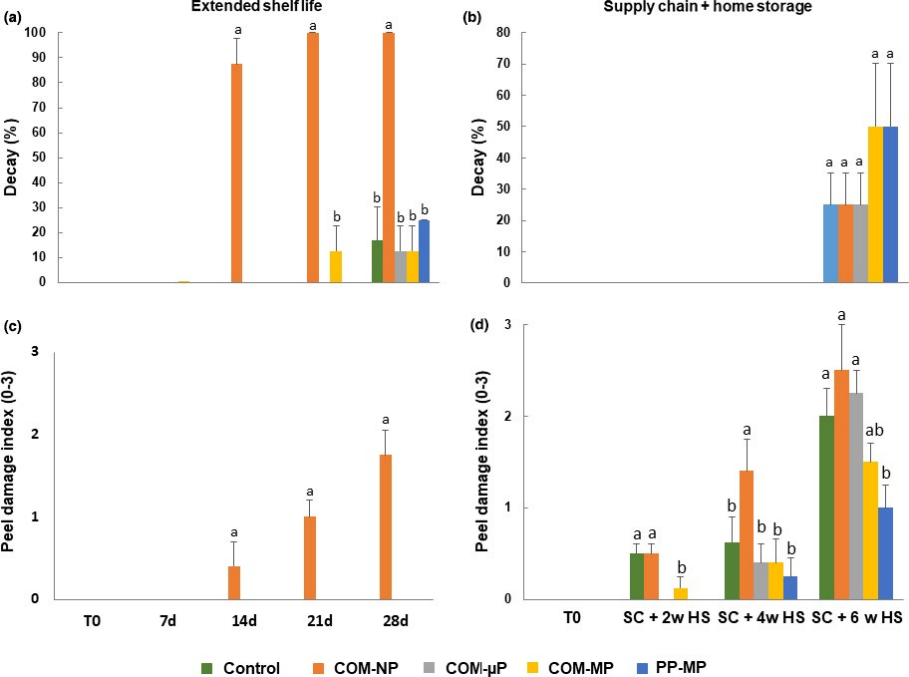
Decay incidence and peel damage indices of red bell pepper fruit in different packages. Measurements were taken during extended shelf at 22°C, and after a simulated supply chain treatment including 2 days at 15°C and 2 days at 22°C followed by up to 6 weeks of refrigerated storage at 4°C. Data are means ± *SE* of 3 replications. Different letters indicate significant differences at *p* ≤ .05. µP, micro‐perforated; COM, compostable; MP, macro‐perforated; NP, nonperforated; PP, polypropylene

After 2 weeks at shelf at 22°C, fruit in the NP packages had a very high rate of 87.5% decay, and decay incidence further reached 100% after 3 and 4 weeks (Figure 6a). In contrast, decay incidence in all other treatments, including the control and all other packed fruit was minor, and was mainly apparent after 4 weeks at the end of the extended shelf life storage period (Figure 6a). At the end of the simulated supply chain storage regime, we observed the presence of 25%–50% decay in all treatments without significant differences among them (Figure 6b).

### 3.5 Peel damage

Peel damage (pitting) can be a result of either chilling injury or CO_2_ damage. During extended shelf at 22°C, we observed significant development of peel damage particularly in the fruit within the NP compostable packages but not in any of the other treatments (Figure 6c). During the supply chain regime, we observed slight but significant peel damage in the control and NP packaging treatments already after 2 weeks of refrigerated storage, and significant higher amounts of peel damage in the NP packages after 4 weeks of refrigerated storage (Figure 6d). After 6 weeks of refrigerated storage, we observed peel damage in all treatments, but the latter were somewhat lower in the PP packages (Figure 6d).

### 3.6 Fruit flavor

**FIGURE 7:**
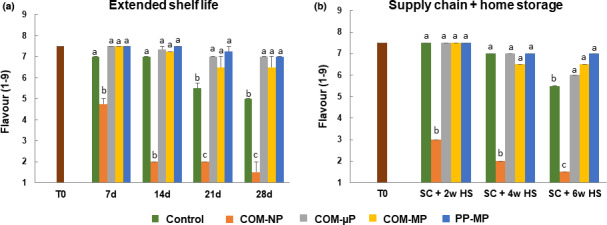
Flavor acceptance scores of red bell pepper fruit in different packages. Measurements were taken during extended shelf at 22°C, and after a simulated supply chain treatment including 2 days at 15°C and 2 days at 22°C followed by up to 6 weeks of refrigerated storage at 4°C. Data are means ± *SE* of 3 replications. Different letters indicate significant differences at *p* ≤ .05. µP, micro‐perforated; COM, compostable; MP, macro‐perforated; NP, nonperforated; PP, polypropylene

The flavor‐acceptance score at the beginning of the experiment was 7.5 (on a scale of 1–9), and it tended to decrease during storage. The most dramatic decline in flavor acceptance has been observed in the NP compostable packages, in which the peppers became inedible (flavor score below 5) already after 1 week of shelf life at 22°C and after the simulated supply chain and 2 weeks of refrigerated storage (Figure 7a,b).

Under continuous shelf at 22°C, the flavor score of the control nonpacked fruit decreased to 7.0 after 1 and 2 weeks, and to 5.5 after 3 weeks, and became inedible after 4 weeks (Figure 7a). The flavor acceptance of the peppers packed in the perforated compostable and PP packages retained better, and after 4 weeks at 22°C the flavor scores in these treatments were between 6.5 and 7.0 (Figure 7a).

At the end of the supply chain simulation (i.e., after 6 weeks of refrigerated home storage), the flavor score of control pepper fruit significantly declined to 5.5, whereas the flavor scores of the peppers packed in the perforated compostable and PP packages remained higher between 6.0 and 7.0 (Figure 7b).

### 3.7 Acceptance score

**FIGURE 8:**
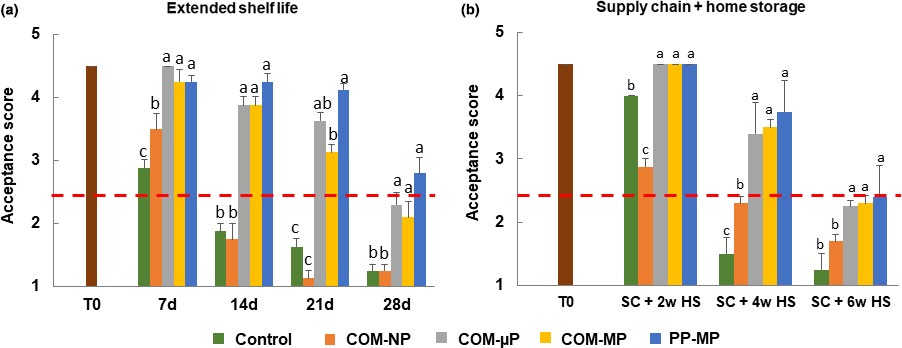
Visual acceptance scores of red bell pepper fruit in different packages. Measurements were taken during extended shelf at 22°C, and after a simulated supply chain treatment including 2 days at 15°C and 2 days at 22°C followed by up to 6 weeks of refrigerated storage at 4°C. Data are means ± *SE* of 3 replications. Different letters indicate significant differences at *p* ≤ .05. µP, micro‐perforated; COM, compostable; MP, macro‐perforated; NP, nonperforated; PP, polypropylene

Fruit acceptance score at harvest was 4.5 (i.e., between *good* and *excellent*). As seen in Figure 8a, during extended shelf at 22°C, the acceptance score of the control nonpacked fruit decreased to 3.5 after 1 week, and became unacceptable (acceptance score below 2.5) after 2 weeks. The acceptance score of the fruit stored in the NP packages also decreased rapidly due to softening and decay, and became unacceptable after 2 weeks (Figure 8a). In distinction, the peppers in the perforated compostable and PP packages remained in good acceptable quality for up to 3 weeks at 22°C. After 4 weeks, the fruit in the PP packages remained above the minimum acceptability limit, but their score was not significantly different from that of the fruit in the µP and MP compostable packages (Figure 8a).

We further observed a decrease in the acceptability of the control nonpacked fruit also along the simulated supply chain regime, as the fruit became unacceptable after 4 weeks of refrigerated storage (Figure 8b). In distinction, the peppers in the perforated compostable and PP packages remained in good quality (acceptance scores between 3.4 and 3.8) after 4 weeks of refrigerated storage (Figure 8b). After 6 weeks of refrigerated storage, the fruit in all perforated packages were slightly below the acceptability limit without any significant differences among them.

## 4 DISCUSSION

The main goals of this research were as follows: (a) to examine if compostable packages may replace the current use of conventional plastic packages for retail marketing of red bell pepper fruit, and (b) to examine what are the preferred perforation levels for these compostable packages. The achieved results indicate that µP and MP compostable packages were very effective in retaining postharvest quality of red bell pepper fruit as examined under two different storage regimes: extended shelf life at 22°C and simulated supply chain, including consequent refrigerated home storage. The main advantages of the perforated compostable packages on retaining postharvest quality of red bell peppers as compared with control nonpacked fruit was demonstrated by reducing weight loss, shriveling and softening, retention of flavor acceptance, and improvement of visual acceptance. The compostable packages did not affect decay development and appearance of peel disorders. These findings are in agreement with previous studies that also reported that the main effect of modified atmosphere packaging on keeping peppers quality was in reducing weight loss and controlling shriveling and softening (Ben‐Yehoshua et al., 1983; Chitravathi et al., 2015; Ilić et al., 2017; Wall & Berghage, 1996).

The perforated compostable packages tested in this study were almost as effective as commercial MP PP packages in retaining pepper fruit quality, despite the fact they have a higher WVTR as compared with PP films, thus resulting in somewhat higher water loss rates during storage (Figure 5a,b). Nonetheless, the compostable packages effectively retained pepper fruit quality and prevented shriveling for up to 3 weeks during extended shelf life at 22°C and for up to 4 weeks of refrigerated storage, in a similar manner to that of the PP packages (Figures 1, 3, and 5c,d). Using compostable films with enhanced water vapor barrier properties would further improve the efficacy of sustainable packaging for bell pepper preservation (Li et al., 2018).

The second question we wanted to address was what are the preferred perforation levels of the compostable packages? In this respect, we found that it is of great importance to perforate the packages, as storage of red bell peppers in NP compostable packages was harmful, causing severe damage manifested by enhanced softening, decay, peel damage, and off‐flavor generation (Figures 2 and 5, 6, 7). Storage of peppers in NP compostable packages resulted in the creation of hypoxic atmosphere in the package headspace characterized by extremely low levels of O_2_ (<1.0%) and high levels of CO_2_ (>10%) (Figure 4). These observed O_2_ and CO_2_ levels are beyond the reported tolerance limits for bell peppers, which are 2% O_2_ and 5% CO_2_ (Cantwell, 2002; Saltveit, 2003). It was reported that exposure of peppers to low O_2_ levels below 1% enhances ethanol fermentation metabolism (Imahori et al., 2002). In agreement with this information, we detected severe fermentative off‐flavors in fruit stored in the NP packages, which became inedible after 1 week at 22°C and after 2 weeks of simulated supply chain and refrigerated storage (Figure 7a,b). It was further reported that exposure of peppers to CO_2_ levels above 5% may cause CO_2_ damage (Cantwell, 2002; Saltveit, 2003), and we have indeed observed severe peel damage on the fruit kept in the NP packages, especially during storage at 22°C (Figures 2 and 6c,d). It is worth notice that the tested compostable packages have a lower OTR value as compared with PP films, and therefore the application of perforations is especially crucial in order to prevent the creation of unfavorable hypoxic conditions. Similar harmful effects of NP compostable packages on fruit quality were reported also for cucumbers (Owoyemi et al., 2021).

Beside the necessity to perforate the compostable packages in order to avoid the development of hypoxic conditions, we have not detected major differences in fruit quality retention between the µP and MP packages. The µP packages tested in this study created a gas atmosphere of 15%–18% O_2_ and 2%–5% CO_2_, whereas the MP packages created a gas atmosphere of 20%–21% O_2_ and 0.1%–0.5% CO_2_ (Figure 4). Ilić et al. (2017) previously examined the efficacy of µP and MP Xtend^®^ polyamide‐based modified atmosphere packages on retaining the quality of mini sweet peppers, and have not found any significant differences between them in terms of weight loss, decay development, and levels of bioactive compounds, but indicated that the µP packages better retarded color change. A recent report indicated that addition of oxygen absorbents to modified atmosphere packages of yellow bell peppers extended shelf life, however, the amount of the oxygen absorber should rather be optimized carefully in order not to lead to the development of anaerobic conditions (Devgan et al., 2019). In case of fresh cut green bell peppers, a modified atmosphere consisting 13%–14% O_2_ and 7% CO_2_ created by Cryovac PD961 films was beneficial for retarding microbial growth (Ranjitha et al., 2015).

## 5 CONCLUSIONS

The findings observed in this study indicate that µP and MP compostable packages were very effective in retaining red bell pepper fruit quality after harvest, and thus may provide an environmentally friendly alternative to the commonly usage of conventional retail plastic packages (Flodberg et al., 2015). Overall, the µP and MP compostable packages extended the life of red bell peppers by 2 weeks, both under continuous shelf life conditions, and during simulated supply chain and refrigerated home storage. In contrast, the use of NP compostable packages was harmful, and enhanced softness, decay and off‐flavor accumulation.

## CONFLICT OF INTEREST

The authors declare that they do not have any conflict of interest.

## ETHICAL APPROVAL

This study does not involve any human or animal testing.

## INFORMED CONSENT

Written informed consent was obtained from all study participants.

## Data Availability

The data that support the findings of this study are available on request from the corresponding author. The data are not publicly available due to privacy or ethical restrictions.
